# Vertebral Osteomyelitis Caused by *Mycobacterium abscessus* Surgically Treated Using Antibacterial Iodine-Supported Instrumentation

**DOI:** 10.1155/2014/197061

**Published:** 2014-12-02

**Authors:** Satoshi Kato, Hideki Murakami, Satoru Demura, Katsuhito Yoshioka, Hiroyuki Hayashi, Noriaki Yokogawa, Xiang Fang, Hiroyuki Tsuchiya

**Affiliations:** Department of Orthopaedic Surgery, Kanazawa University School of Medicine, 13-1 Takara-machi, Kanazawa 920-8641, Japan

## Abstract

*Mycobacterium abscessus* infections rarely develop in healthy individuals, and mostly they occur in immunocompromised hosts. Vertebral osteomyelitis due to *Mycobacterium abscessus* is very rare and only three previous cases of spinal infection caused by *Mycobacterium abscessus* have been reported. *Mycobacterium abscessus* isolates are uniformly resistant to antituberculous agents and can display a virulent biofilm-forming phenotype. The patient was a 67-year-old woman with vertebral osteomyelitis of the L1-2. She was healthy without immune-suppressed condition, history of trauma, or intravenous drug use. The smear examination of the specimen harvested by CT-guided puncture of the paravertebral abscess revealed *Mycobacterium abscessus*. Her disease condition did not abate with conservative treatment using antimicrobial chemotherapy. Radical debridement of the vertebral osteomyelitis and anterior reconstruction from T12 to L2 using antibacterial iodine-supported instrumentation were performed. Chemotherapy using clarithromycin, amikacin, and imipenem was applied for 6 months after surgery as these antibiotics had been proven to be effective to *Mycobacterium abscessus* after surgery. Two years after surgery, the infected anterior site healed and bony fusion was successfully achieved without a recurrence of infection.

## 1. Introduction

*Mycobacterium abscessus*, a rapidly growing mycobacterium, is ubiquitous in soil and aqueous environments, including municipal drinking water and sewage systems [1, 2]. The most common clinical presentations of diseases caused by* Mycobacterium abscessus* are pulmonary, localized skin and soft tissue infection, as exemplified by the predominant mycobacteria causing nosocomial surgical site infection [2].* Mycobacterium abscessus* infections rarely develop in healthy individuals and are substantially more common in immunocompromised hosts [3]. In the present case report, we describe a healthy 67-year-old woman with vertebral osteomyelitis in the lumbar spine caused by* Mycobacterium abscessus*. The patient was successfully treated with adequate chemotherapy and radical debridement and spinal reconstruction using antibacterial iodine-supported instrumentation.

## 2. Case Report

**Figure 1:**
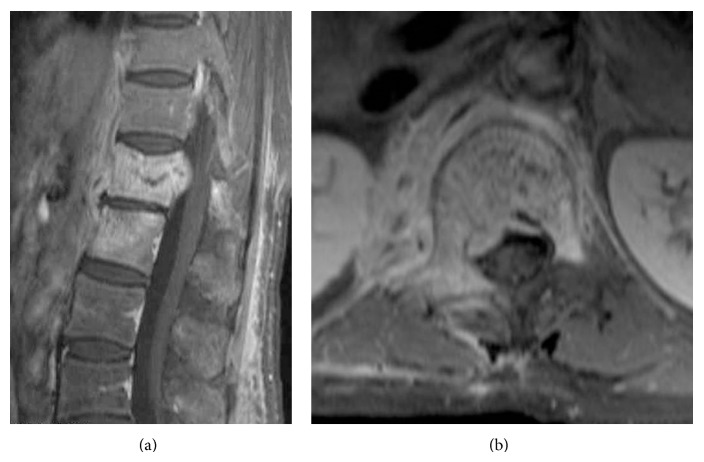
Enhanced T1-weighted magnetic resonance imaging showing vertebral osteomyelitis which involved the L1 and L2 vertebral bodies. (a) Sagittal view. (b) Axial view.

A 67-year-old woman, with compensated hypothyroidism and hypertension, presented to another hospital with continuous low back pain and a low-grade fever of 3-month duration. She was diagnosed with subacute thyroiditis and treated accordingly; however, her symptoms did not abate. Computed tomography (CT) and magnetic resonance imaging (MRI) of the lumbar spine revealed destructive changes of the L1 and L2 vertebral bodies; these changes were surrounded by paravertebral abscesses (Figure 1). A culture examination of the specimen harvested by CT-guided puncture of the paravertebral abscess revealed nontuberculous mycobacteria identified as* Mycobacterium abscessus*, which were noted to be resistant to antituberculous agents in vitro. The patient's pain level and spinal destruction gradually advanced. Additionally, there was a continuous discharge of pus from the puncture hole despite chemotherapy treatment with ethambutol, rifampicin, and isoniazid. The patient presented to our hospital 6 weeks after the diagnosis.

Upon admission to our hospital, the patient had significant back pain without bladder or bowel dysfunction, or neurologic deficit of the lower extremities. Laboratory findings showed a white blood cell count of 4310/*μ*L, C-reactive protein of 2.7 mg/dL, and an erythrocyte sedimentation rate of 53 mm/h. A chest CT showed no signs indicative of pulmonary infection. We changed the patient's regimen of antibiotics to include clarithromycin (800 mg per day, 400 mg orally twice daily), amikacin (600 mg per day, 300 mg intravenously twice daily), and imipenem (2000 mg per day, 1000 mg intravenously twice daily) for 3 weeks after admission to our hospital. However, her condition did not improve, and the continuous discharge of pus did not change. Smear examination of the pus discharged from the puncture hole did not reveal any types of bacteria.

**Figure 2:**
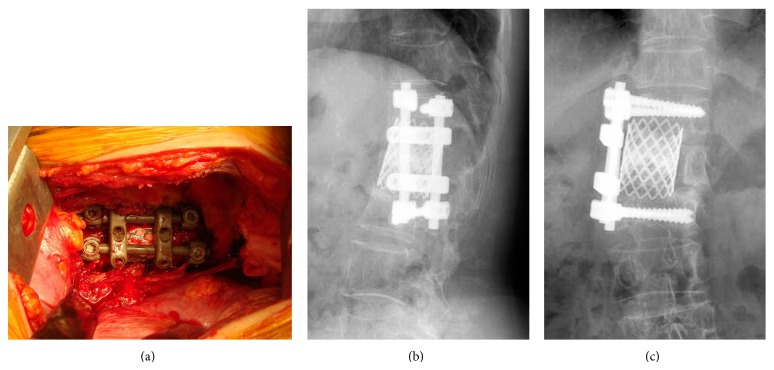
Anterior reconstruction from T12 to L2 using iodine-supported instrumentation. (a) Operative photograph. (Right side is directed cranially, and upper side is directed dorsally.) Postoperative radiographs of the lumbar spine. (b) Lateral view. (c) Anteroposterior view.

**Figure 3:**
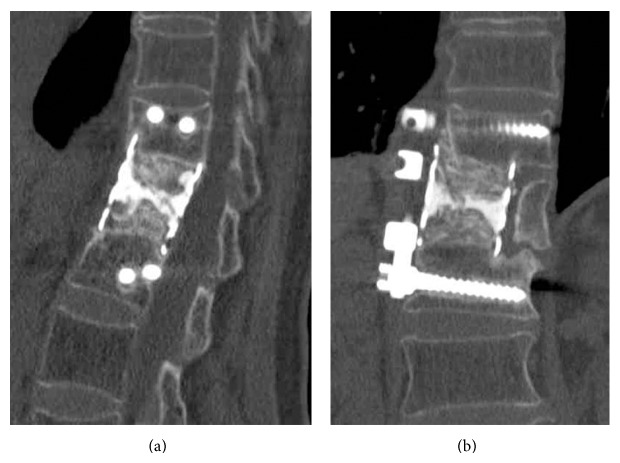
Computed tomography of the lumbar spine 2 years after surgery showing a bony fusion was successfully achieved without a recurrence of infection. (a) Sagittal view. (b) Coronal view.

Radical debridement of the vertebral osteomyelitis of the L1 and L2 vertebral bodies and spinal reconstruction using iodine-supported instrumentation were performed using a right retroperitoneal approach. An autogenous bone graft harvested from the iliac crest and alpha-TCP paste mixed with imipenem were packed into an iodine-supported mesh cage. The mesh cage was inserted into the large defect after a radical debridement involving L1 corpectomy. Then, anterior fixation from T12 to L2 using antibacterial iodine-supported instrumentation was performed (Figure 2). Antimicrobial chemotherapy using clarithromycin for 6 months and amikacin and imipenem for 3 months at the same dose of the preoperative period was continued after surgery, as these antibiotics have been proven to be effective against* Mycobacterium abscessus*, which was identified on the culture examination of the specimen harvested during surgery. A hard brace was applied for 3 months after surgery. Laboratory findings at 3 months after surgery showed no evidence of inflammatory signs; white blood cell count was 5000/*μ*L, C-reactive protein level was 0.1 mg/dL, and erythrocyte sedimentation rate was 4 mm/h. Two years after surgery, the infected anterior site had healed, and bony fusion was successfully achieved (Figure 3). The patient is now completely asymptomatic without a brace and has not had a recurrence of infection. No evidence of inflammatory signs has been apparent in subsequent laboratory examinations.

## 3. Discussion

Nontuberculous mycobacteria infections of the musculoskeletal system are uncommon and mostly occur in immunocompromised patients. A search of the literature revealed only 3 cases of spinal infection caused by* Mycobacterium abscessus* [4–6]. One of these patients had been receiving corticosteroid therapy for systemic lupus erythematosus [4]. Trauma has been reported as a major predisposing factor for osteomyelitis caused by nontuberculous mycobacteria. Another patient had a history of blunt trauma to the back [5], and the last patient had a history of intravenous drug abuse [6]. The case presented in this paper was of a healthy woman without an immune-suppressed condition and no history of trauma or intravenous drug use. However, because of its rarity, the treatment protocol of nontuberculous mycobacteria infections of the musculoskeletal system has not been established according to subspecies. The antimicrobial regimens used in nontuberculous mycobacteria differ from those used against* Mycobacterium tuberculosis*. In general, nontuberculous mycobacteria are more resistant to antituberculous agents.* Mycobacterium abscessus* isolates are uniformly resistant to the standard antituberculous agents [7]. The choice of an effective antibiotic is further complicated by the fact that clarithromycin, amikacin, imipenem, and cefoxitin are the only antibiotics that are reliably active against* Mycobacterium abscessus* [8]. According to the American Thoracic Society, drug therapy or combined surgical and medical therapy is recommended for nonpulmonary nontuberculous mycobacterium infection [8]. Prolonged therapy in combination with surgical debridement is strongly recommended for all cases of vertebral osteomyelitis caused by nontuberculous mycobacteria, especially in patients with abscess formation [8, 9].

In the last decade, the use of anteriorly applied metallic implants within the infected area has been debated [10–12]. The anterior implantation of a titanium mesh cage, filled with an autogenous bone graft and supplemented by anterior or posterior rigid fixation, has advantages in the course of infection healing by providing rigid stabilization of unstable vertebrae after radical debridement. However, there is still controversy regarding the use of a metallic implant within the infected area. In vitro studies showed that biofilm, which is adherent to metallic implants, renders them invulnerable to antibiotics [13].* Mycobacterium abscessus* can display a smooth biofilm-forming phenotype and an ability to switch between smooth and rough morphologies allowing it to transition between a colonizing phenotype and a more virulent, invasive form [14].

In our institute, antibacterial iodine-supported titanium implants have been developed and have been observed in a clinical trial. Iodine-supported titanium implants, with iodine-containing surfaces that were modified using anodization, had antibacterial activity, biocompatibility, and no cytotoxicity [15]. The results of the clinical trial demonstrated that iodine-supported implants can be effective in the prevention of infections after orthopedic surgery and the surgical treatment of musculoskeletal infections [16]. In the case presented here, the rigid spinal fixation achieved by using this antibacterial implant provided early mobilization and excellent bony fusion without a recurrence of infection. Iodine-supported instrument can be a good option of surgical implants for vertebral osteomyelitis.

## 4. Conclusion

We present a rare case of vertebral osteomyelitis caused by* Mycobacterium abscessus*. The patient was successfully treated by adequate chemotherapy and radical debridement and spinal fusion using antibacterial iodine-supported instrumentation.
